# Therapeutic Notes

**Published:** 1891-03

**Authors:** 


### Therapeutic Notes.

---

### THE ELECTRIC LIGHT AS A THERAPEUTIC AGENT.

In the *Revista de Ciencias Medicas*, Dr. Estanislao von Stein reports a number of neurasthenic, hysterical, and rheumatic affections successfully treated by illuminating the surfaces with the electric light. His apparatus — "Fotoforo" — consists of an incandescent light of twelve volts, supplied with an infundibuliform reflector and a handle. Illuminating a painful joint or nerve, as sciatica, for two to five minutes, has yielded him surprising results. The alterations, especially of the nerves, which takes place are probably of a molecular character.

---

Dr. Kianouski, in an article on the Microbicide Action of the Gastric Juice, comes to the following conclusions: The empty stomach of a healthy man contains innumerable organisms. The gastric juice, and principally the hydrochloric acid, possesses microbicide properties. The microbes take no active part in digestion. Persons who, on account of some affections, secret little hydrochloric acid, are easily intoxicated, by means of the microorganisms in the stomach. Therefore, the stomach should not remain in an empty condition for any length of time, and during an epidemic, food should be taken at frequent intervals, and, if possible, sterilized.

---

On the action of caffeine, morphine, atropine, ergot, and digitalis upon arterial pressure, by Dr. S. Frenkel, in *Deutsches Archiv. f. Klin. Medicin*, by means of Basch's sphygmograph, the author was enabled to arrive at the following conclusions: Caffeine, administered internally, 50–80 centigrammes daily, increases arterial pressure, and acts like digitalis. Hypodermic injection, 10–60 c.g., raises the intravascular pressure rapidly, and is consequently indicated in collapse due to cardiac disease. Morphine, injected hypodermically, 1–3 c. g., increases the intravascular pressure slightly. Frequently, however, this effect is not noted. Theoretically, therefore, morphine is not contra-indicated in cardiac affections. Atropine, injected hypodermically $\frac{3}{16}$–1 milligram, elevates the arterial tension from 20 to 25 millimeters. The pulse is accelerated. No change is observed in the quantity of urine passed. Ergotine, after an interval of two to three hours, provokes an elevation of arterial pressure from 20 to 30 millimeters. Generally the pulse is rendered slower but more forcible. Digitalis, given to patients suffering with cardiac affections, has yielded classical results analogous to those obtained when injected hypodermically in animals.

---

Up to December 9th, Professor Dujardin-Beaumetz, of Paris, had inoculated five cases of tuberculosis, with results in conformity with Koch's assertions. From the experiences of such investigators as Dujardin-Beaumetz, the medical world should wait before drawing conclusions.—*Revue General de Therapeutique.*

---

Dr. Pean's results with Koch's lymph (*Le Progrès Médical*, December 13th,) are summed up as follows: Notable amelioration of local lesions; suppurations tend to diminish; fistulous tracts are closing; ulcerations are granulating nicely; but, as yet, no real cure has taken place.

---

Before the Academy of Medicine, of Paris, Dr. Boisseau du Rocher read a paper on Internal Franklinisation in Neurasthenia. In dilatation of the stomach, excellent results have been obtained by the production of ozone and by stimulating the secretions. Furthermore, the electric charge penetrates the tissues, keeping them at their maximum intensity under a constant electric pressure, thus reëstablishing the equilibrium of the nervous system.—*Le Progrès Médical.*

---

In the *Bulletin Général de Therapeutique* are given the following formula for using Iodol, the new antiseptic:

| SOLUTION. | | INHALATION. | |
|---|---|---|---|
| Iodol.................. | 1 part. | Iodol................. | 1 part. |
| Alcohol, 95 per cent.,.. | 16 parts. | Alcohol................ | 15 parts. |
| Glycerine............. | 34 parts. | Glycerine............. | 10 parts. |
| | | Water................ | 10 parts. |

| COLLODION. | | POMMADE. | |
|---|---|---|---|
| Iodol | 1 part. | Iodol | 2 grammes. |
| Ether | 10 parts. | Vaseline | 10 grammes. |
| Gun cotton | 5 parts. | | |

POMMADE.

| | |
|---|---|
| Iodol | 5-10 grammes. |
| Lanoline | 100 grammes. |

---

In a man suffering from well-marked typhoid fever of ten days' standing, a writer in the *Lancet*, June 21st, states that, thinking this a good case in which to try the remedy, he sent the man home to bed, and ordered him *b*-naphthol in four-grain doses every third hour. The medicine was given in capsules. His symptoms, which were very severe and well marked, at once began to ameliorate, and he was soon convalescent. He adds, there can be no doubt about the marked good effect of naphthol in typhoid, as also in Summer diarrhea and dysentery.—*Canada Medical Record.*

---

In *Northwestern Medical Journal*, Dr. Crone treated fourteen out of eighteen cases of diphtheria successfully as follows: Quinia, sulphur and boric acid, equal parts, insufflated every two hours. The following day the swelling had partially subsided, and the membrane began to disappear. Convalescence in from five to seven days. In addition to this treatment pot. chlor. and tinct. ferri chlor. and brandy were administered.

---

Two drops of creasote made from beech tar, given with a little water, is said to be a specific for hiccough arising from drunkenness.—*Lancet-Clinic.*

---

When a silver catheter is blackened by the urine, it denotes the presence of pus in the bladder, which generates sulphuretted hydrogen.

---

For the treatment of eczema, a writer in the Cincinnati *Lancet-Clinic* advises an ointment composed of oil of pine tar, ʒj.; vaseline, ℥j.

